# Sulfur-Oxidizing Bacteria Alleviate Salt and Cadmium Stress in Halophyte *Tripolium pannonicum* (Jacq.) Dobrocz.

**DOI:** 10.3390/ijms25052455

**Published:** 2024-02-20

**Authors:** Aleksandra Koźmińska, Iwona Kamińska, Ewa Hanus-Fajerska

**Affiliations:** Department of Botany, Physiology and Plant Protection, Faculty of Biotechnology and Horticulture, University of Agriculture in Krakow, Al. Mickiewicza 21, 31-120 Cracow, Poland; i.kaminska@urk.edu.pl (I.K.); e.hanus-fajerska@urk.edu.pl (E.H.-F.)

**Keywords:** salt stress, cadmium stress, halophytes, bioinoculant, sulfur-oxidizing bacteria, stress markers, soil inoculation

## Abstract

The aim of this study was to investigate how introducing halophilic sulfur-oxidizing bacteria (SOB) *Halothiobacillus halophilus* to the growth substrate affects the physiological and biochemical responses of the halophyte *Tripolium pannonicum* (also known as sea aster or seashore aster) under salt and cadmium stress conditions. This study assessed the plant’s response to these stressors and bacterial inoculation by analyzing various factors including the accumulation of elements such as sodium (Na), chloride (Cl), cadmium (Cd) and sulfur (S); growth parameters; levels of photosynthetic pigments, proline and phenolic compounds; the formation of malondialdehyde (MDA); and the plant’s potential to scavenge 2,2-Diphenyl-1-picrylhydrazyl (DPPH). The results revealed that bacterial inoculation was effective in mitigating the deleterious effect of cadmium stress on some growth criteria. For instance, stem length was 2-hold higher, the growth tolerance index was 3-fold higher and there was a 20% increase in the content of photosynthetic pigments compared to non-inoculated plants. Furthermore, the SOB contributed to enhancing cadmium tolerance in *Tripolium pannonicum* by increasing the availability of sulfur in the plant’s leaves, which led to the maintenance of an appropriate, about 2-fold-higher level of phenolic compounds (phenylpropanoids and flavonols), as well as chloride ions. The level of MDA decreased after bacterial application in all experimental variants except when both salt and cadmium stress were present. These findings provide novel insights into how halophytes respond to abiotic stress following inoculation of the growth medium with sulfur-oxidizing bacteria. The data suggest that inoculating the substrate with SOB has a beneficial effect on *T. pannonicum*’s tolerance to cadmium stress.

## 1. Introduction

In the era of a rapidly changing climate, plants are exposed to enormous environmental pressure, which has a negative impact on biomass production, and in the case of crop production, is leading to a global economic crisis. Moreover, soil salinity and its contamination with different heavy metals are among the main factors deepening degradation of the soil environment and negatively affecting the growth and development of plants worldwide [1,2,3]. Fortunately, some halophytic plant species have developed physiological, biochemical and molecular adaptation mechanisms for such conditions [4]. Halophytes are recognized for their capability to thrive in concentrations of sodium and chloride ions that would be detrimental to the majority of crop species [5]. The adaptation mechanisms enabling halophytes to survive high salt concentrations may also confer tolerance to other toxic ions, including heavy metals, such as cadmium, because some of the mechanisms are compatible for these two stressors [6,7,8]. Those mechanisms include control of ion homeostasis, accumulation of osmolytes, activation of antioxidant systems and production of osmoprotectants [9,10,11].

Recent genetic and molecular research has revealed complicated regulatory pathways and networks through which halophytes coordinate their adaptation and tolerance to stress [12,13,14,15]. Numerous molecules are involved in these networks, with sulfur (S) being one of the key players. Amino acids and metabolites containing sulfur maintain mechanisms within plant cells to enhance stress tolerance. They interact with various biomolecules like plant hormones, polyamines, nitric oxide (NO) and even with other nutrients in plants, producing essential derivatives crucial for resilience against abiotic stressors [16,17]. Sulfur and its derivatives such as glutathione (GSH), hydrogen sulfide (H_2_S), methionine (Met), cysteine (Cys), phytochelatin (PC), ATP sulfurylase (ATPS) and protein thiols have been documented to enhance the antioxidant defense needed to eliminate excessive reactive oxygen species (ROS) under different abiotic stresses. Besides the direct impact, the signaling role of sulfur and interaction with other molecules also work to counter abiotic stress [18,19,20,21,22,23,24,25,26].

Unfortunately, we often encounter sulfur deficiency in agricultural soils around the world, which is one of the factors limiting the stress tolerance of many plants [27]. Moreover, the majority of sulfur in soil (>95% of total sulfur) is bound to organic compounds, making it inaccessible to plants directly. To remedy this situation, utilizing sulfur-oxidizing bacteria (SOB) boosts the pace of sulfur oxidation and accelerates sulfate production [28,29]. This process renders sulfur better available to plants and consequently causes an increase in S-containing compounds (such as GSH, methionine, thioredoxins, vitamins, coenzyme A or GSH-associated antioxidant enzymes), which has already been noted to strengthen the antioxidant defense system in plants under exposure to various stresses including salinity or cadmium. Environmental applications of SOB encompass hydrogen sulfide detoxification, soil bioremediation and wastewater treatment. SOB capable of producing elemental sulfur are implicated in biological sulfur soil treatments, such as the restoration of saline alkali soils and the oxidation of minerals containing sulfide [30,31,32,33]. Recently, the utilization of sulfur-oxidizing bacteria as agents promoting plant growth has also been obtaining traction.

We now know that some growth-promoting bacteria can improve the growth of several halophytes [34,35]. The inoculation of plants with sulfur-oxidizing bacteria (SOB) has already been reported [36,37,38,39], but there is still a knowledge gap on the impact of inoculation of saltmarsh eudicots with specific SOB isolates.

Given the established correlation between *T. pannonicum*’s reaction to salt or cadmium stress and alterations in its antioxidant system [8], exploring the effects of SOB soil inoculation, which may enhance sulfur levels and thus specific antioxidants, on *T. pannonicum*’s response to these stressors is warranted.

Hence, the main aim of this research was to examine the impacts of introducing halophilic sulfur-oxidizing bacteria (SOB) *Halothiobacillus halophilus* DSM 6132 to the growth substrate, to investigate the physiological and biochemical reactions of the halophyte *T. pannonicum* under salt and cadmium stress. Sea aster (*Tripolium pannonicum* (Jacq) Dobrocz., also known as *Aster tripolium* L.) is a perennial halophyte indigenous to Eurasia and northern Africa, confined in its habitats to salt marshes, estuaries and occasionally inland saline regions. It thrives in well-drained lightweight, medium and heavy soils with a broad spectrum of pH levels. This plant species has garnered attention as a promising commercial vegetable due to its subtle salty taste and significant nutritional value, making it a novel type of food [40,41]. *T. pannonicum* has also been identified as a model plant for studying how plants tolerate environmental stresses [42]. Although we know how *T. pannonicum* copes with salt stress, there are limited reports on its reaction to heavy metals [8,43,44] and the underlying mechanisms of these responses remain inadequately elucidated.

We hypothesized that soil inoculation with SOB would increase the pool of phytoavailable sulfur and induce beneficial changes in the response of *Tripolium pannonicum* to applied stresses. We also assumed that introducing sulfur-oxidizing bacteria to the growing medium aimed at cultivating plants would enhance the adaptation of *Tripolium pannonicum* to the stresses induced by salt and cadmium.

## 2. Results

### 2.1. Effect on Growth Parameters

**Figure 1:**
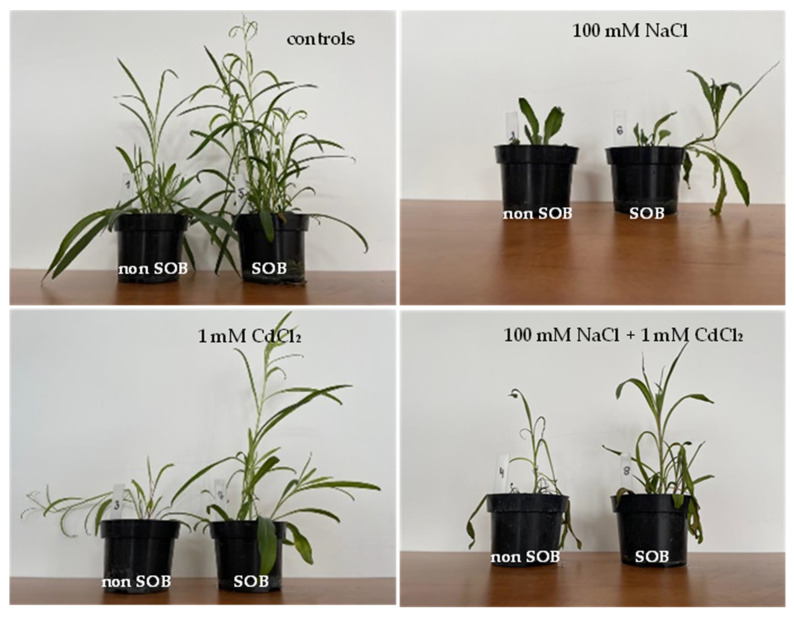
Morphology of *Tripolium pannonicum* exposed to abiotic stresses and cultivated on medium inoculated with *Halothiobacillus halophilus* (SOB) and not inoculated with *H. halophilus* (non-SOB).

*Tripolium pannonicum* plants subjected to salinity grew shorter than control and cadmium-stressed plants, of approx. 70–80% and 50–65%, respectively. Inoculation of the substrate with SOB caused a 2-fold increase in the lengths of the shoots of the tested plants (Figure 1) and an approx. 3-fold increase in the value of the growth tolerance index (GTI) in the leaves and roots of the plants treated with CdCl_2_ (Table 1).

The applied stresses resulted in an inhibition of stem growth in both inoculated and non-inoculated plants; however, the addition of SOB minimized this inhibition in the case of cadmium stress.

### 2.2. Effect on Biochemical Parameters

#### 2.2.1. Sulfur, Sodium, Chloride and Cadmium Absorption by *Tripolium pannonicum* Plants

**Figure 2:**
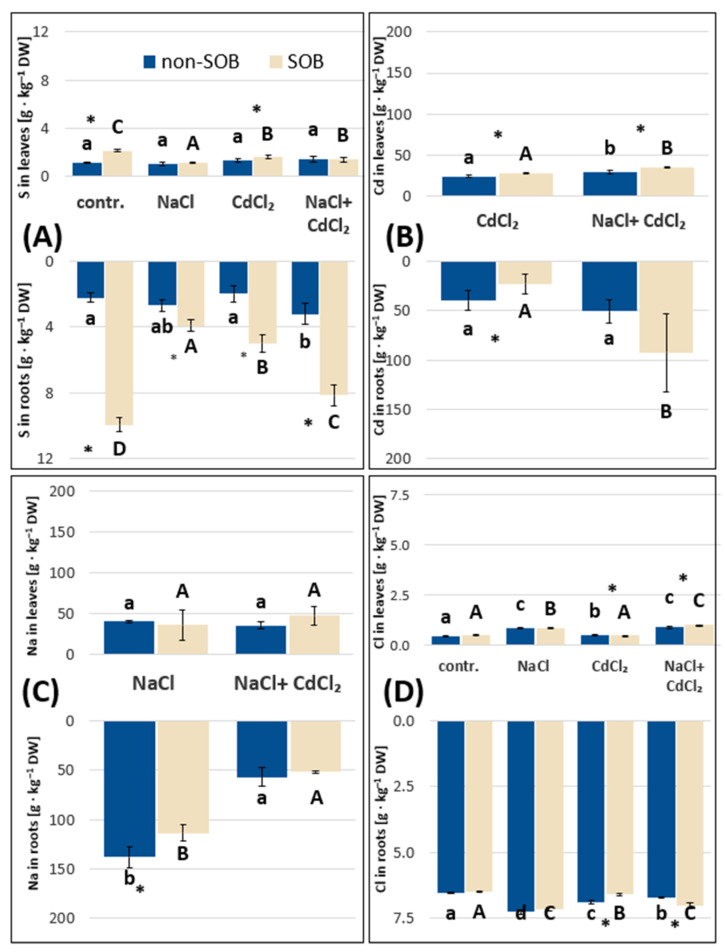
Elemental contents: sulfur (**A**), cadmium (**B**), sodium (**C**) and chloride (**D**) in leaves and roots of *T. pannonicum* subjected to sodium chloride (100 mM NaCl), cadmium chloride (1 mM CdCl_2_), a mixture of sodium chloride and cadmium chloride (100 mM NaCl + 1 mM CdCl_2_) and sulfur-oxidizing bacteria. SOB—substrate inoculated with *Halothiobacillus halophilus*, non-SOB—substrate not inoculated with *H. halophilus*. Different lowercase letters indicate significant differences between plants cultivated on substrate non-inoculated by SOB within different stress treatments. Different capital letters indicate statistically significant differences between plants cultivated on substrate inoculated by SOB within different stress treatments. * indicates statistically significant differences between inoculated and non-inoculated plants within the same stress treatment, according to Tukey’s test (α = 0.05), ±SE, *n* = 5.

The applied stresses did not change the sulfur contents in leaves compared to the control in the non-SOB variant. SOB application increased sulfur contents in plant roots in all four variants of the experiment (by 3-fold on average) and, in turn, an increase in sulfur contents in leaves was noted in the group of control and CdCl_2_ treated plants (Figure 2A). Cadmium accumulation within the roots of non-SOB plants was at the same level in both cadmium treatments, but Cd deposition in the leaves was slightly higher in plants exposed to NaCl + CdCl_2_ (Figure 2B). SOB inoculation caused significantly lower accumulation of Cd in the roots of CdCl_2_-treated plants, but in the case of leaves, significantly higher concentrations of Cd were noted in both cadmium treatments. The addition of SOB did not change the levels of sodium ions in the leaves and it reduced the Na contents in the roots of plants treated with NaCl (Figure 2C). The contents of chlorine ions increased slightly under SOB in the leaves and roots of plants treated simultaneously with NaCl and CdCl_2_ and decreased in plants treated with CdCl_2_ only. The contents of chloride were much higher (~3 fold) in roots than in leaves in all four variants of the experiment, regardless of bacterial inoculation (Figure 2D). It is worth noting that sulfur, cadmium, sodium and chlorine accumulated mainly in the roots (Figure 2).

#### 2.2.2. Photosynthetic Pigments

**Figure 3:**
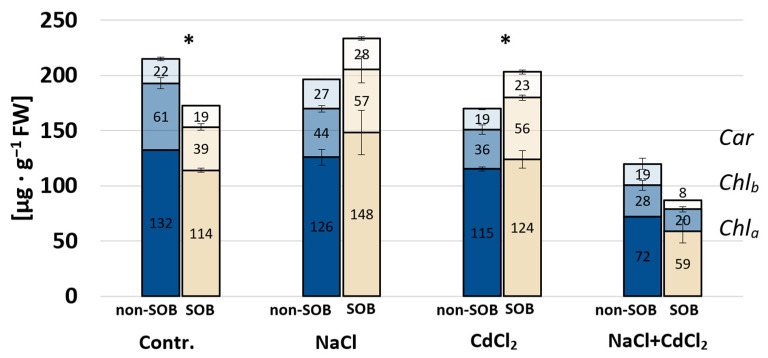
Photosynthetic pigments: carotenoids (Car), chlorophyll a (Chla) and chlorophyll b (Chlb) in *T. pannonicum* subjected to sodium chloride (100 mM NaCl), cadmium chloride (1 mM CdCl_2_), a mixture of sodium chloride and cadmium chloride (100 mM NaCl + 1 mM CdCl_2_) and sulfur-oxidizing bacteria. SOB—substrate inoculated with *Halothiobacillus halophilus*, non-SOB—substrate not inoculated with *H. halophilus*. * indicates statistically significant differences between inoculated and non-inoculated plants within the same stress treatment, according to Tukey’s test (α = 0.05), ±SE, *n* = 5.

The highest content of photosynthetic pigments (233 mg·g^−1^) was recorded in plants cultivated on SOB and under salt stress, and in plants subjected to both sodium and cadmium chloride salts, the content was the lowest (Figure 3). In control plants, a 20% lower content of total photosynthetic pigments was measured when SOB was present in the soil. A significant increase, by approx. 20%, in the total content of photosynthetic pigments after soil bacterial inoculation was recorded only under CdCl_2_ stress conditions, but in the case of NaCl treatment, a similar tendency was noted (though not statistically significant).

#### 2.2.3. Proline, Phenolic Compounds, DPPH Radical and Lipid Peroxidation

**Figure 4:**
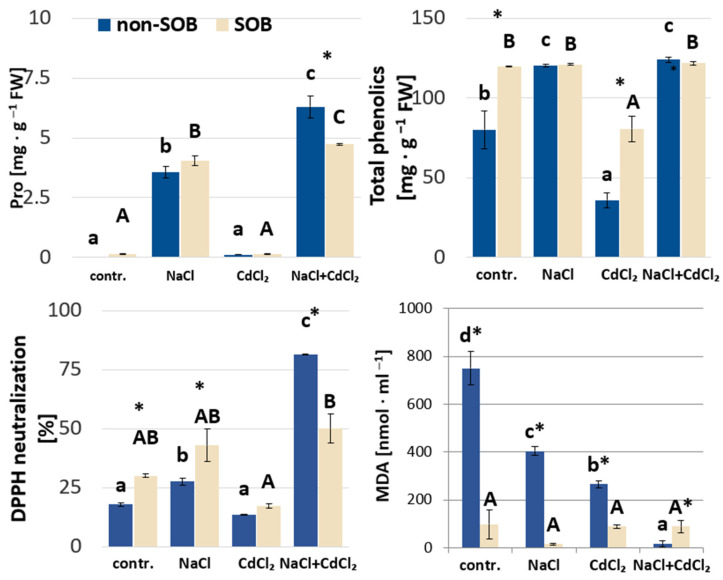
Biochemical parameters in *T. pannonicum* subjected to sodium chloride (100 mM NaCl), cadmium chloride (1 mM CdCl_2_), a mixture of sodium chloride and cadmium chloride (100 mM NaCl + 1 mM CdCl_2_) and sulfur-oxidizing bacteria. SOB—substrate inoculated with *Halothiobacillus halophilus*, non-SOB—substrate not inoculated with *H. halophilus*. Different lowercase letters indicate significant differences between plants cultivated on substrate non-inoculated by SOB within different stress treatments. Different capital letters indicate statistically significant differences between plants cultivated on substrate inoculated by SOB within different stress treatments. * indicates statistically significant differences between inoculated and non-inoculated plants within the same stress treatment, according to Tukey’s test (α = 0.05), ±SE, *n* = 5.

Interestingly, the proline (Pro) content proved to be significantly higher in plants treated with sodium chloride (NaCl and NaCl + CdCl_2_ variants) in comparison to the control and the CdCl_2_ variant of the experiment, where a very low content of this amino acid was recorded (only 0.1–0.15 mg·g^−1^ FW) (Figure 4). A significantly lower Pro content in the leaves of *T. pannonicum* with SOB was observed in plants treated simultaneously with NaCl and CdCl_2_.

*Tripolium pannonicum* reacted to salinity stress with a 70% higher accumulation of phenolic compounds in comparison with control plants (Figure 4), but when treated only with CdCl_2_, over 40% lower values of those substances were noted. Soil inoculation with *Halothiobacillus halophilus* resulted in significantly higher contents of total phenolic compounds in control plants and plants treated with CdCl_2_, exceeding the corresponding value of the non-inoculated variant by 0.5- and 2-fold, respectively. Addition of SOB to the substrate for plants stressed with NaCl and NaCl + CdCl_2_ did not change the concentration of phenolic compounds, but in those variants, phenolic concentrations were found to be relatively high (Table 2). Thus, regardless of SOB inoculation, the level of total phenols was found to be fairly consistent in plants treated with NaCl and the mixture of NaCl + CdCl_2_.

Our analysis of the phenolic compound profile revealed that in *T. pannonicum* leaves, the most abundant phenolics were phenylpropanoids (regardless of treatments used), reaching 36–56% of total phenolics, followed by flavonols (20–43%) and anthocyanins (1.3–8.5%). In all tested plants, the phenylpropanoid: flavonol ratio was about 2:1, except for in CdCl_2_-stressed plants, where a 1.3:1 ratio was recorded. Under the influence of cadmium stress (CdCl_2_ and NaCl + CdCl_2_), the addition of SOB caused a higher content of phenylpropanoids in *T. pannonicum* plants (by approx. 60% and 24%, respectively) (Table 2). The differences in flavonol contents in the leaves among the tested plants (all stresses and SOB treatments used) corresponded with those noted for phenylpropanoids. As for anthocyanins, their levels were quite low in all plants.

The radical scavenging activity, expressed by the high proportion of neutralized DPPH radical (1,1,-diphenyl-2-picrylhydrazyl), was found to be high (over 75%) in plants stressed by NaCl + CdCl_2_. In plants of other tested variants, DPPH˙ neutralization was around 25%. SOB inoculation caused an increase in scavenging activity in control and NaCl-treated plants. The opposite trend was observed with two simultaneous loadings of NaCl and CdCl_2_, where inoculation of sulfur-oxidizing bacteria resulted in lower DPPH neutralization (down to 50%).

The level of malondialdehyde (MDA), a product of membrane lipid peroxidation and a reliable biomarker of oxidative stress, was significantly lower after soil SOB inoculation by about 7.5-, 25- and 3-fold in the control, NaCl and CdCl_2_ variants, respectively. Interestingly, the highest level of MDA was recorded in control plants cultivated on the substrate without the addition of SOB, which was higher than that for stressed plants without SOB.

### 2.3. Multivariate Analysis

**Figure 5:**
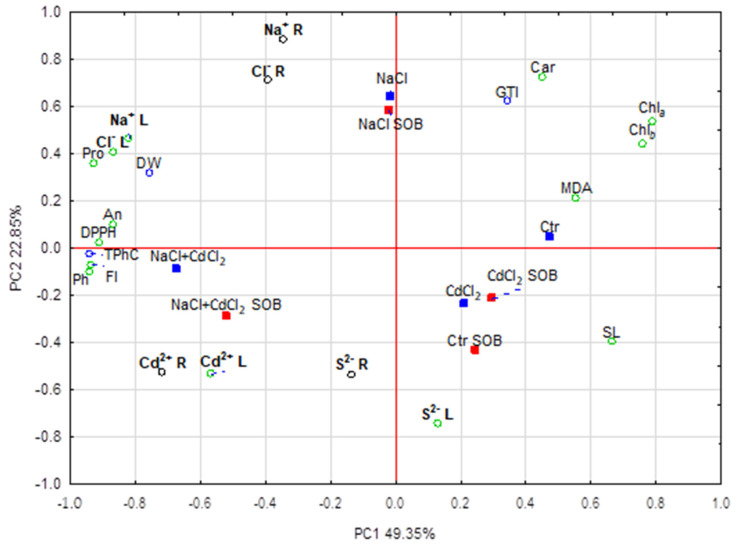
PCA results—scatterplot of the loading factors. Color coding for the presented data is as follows: green circles—parameters measured in above-ground parts (L—leaves); brown circles—parameters measured in the roots (R—roots); blue squares—non-SOB treatments; red squares—SOB treatments; abbrev.: Chla—chlorophyll a, Chlb—chlorophyll b, Car—carotenoids, GTI—growth tolerance index, DW—dry weight, SL—stem length, Pro—proline, DPPH—RSA with DPPH˙, TPhC—total phenolic compounds, Ph—phenylpropanoids, Fl—flavonols, An—anthocyanins, Cd—cadmium, S—sulfur, Na—sodium, Cl—chloride (all elements are in bold).

The first two principal components, PC1 (41.4%) and PC2 (19.9%), explained 61.3% of the total variation. The proline and phenolic substances, chlorophyll content, RSA and leaves’ sodium and chloride accumulation were the most highly correlated variables with PC1. The addition of PC2 enabled a visualization of changes in the sodium, chloride and cadmium accumulation in the roots, along with the sulfur and carotenoid contents within leaf tissues. PC1 discriminated plants growing in the control and multi-stress conditions, whereas PC2 discriminated plants under saline conditions. No strong discrimination between plants cultivated on the substrate inoculated with SOB and the non-inoculated substrate was observed (Figure 5).

## 3. Discussion

Abiotic stressors are known to cause serious problems in agriculture mainly by significantly reducing the yields of cultivated plants. In this context, the metabolic adaptive responses of plants under such stressors are of great interest [45,46]. One of the key players in this regard is sulfuric substances. Plants absorb sulfates, an oxidized form of elemental sulfur (S°), from the soil [47].

Especially under salinity and heavy metal stress, demand for sulfate for plants increases, reflecting the important role of sulfur-containing metabolites in the acquisition of plant tolerance, which has been widely reported [22,23,24,48,49,50]. In recent years, the importance of sulfur-containing compounds in plants’ tolerance to stressful abiotic factors has been well-recognized due to the common occurrence of sulfur deficiencies in soil around the world, as well as the form in which it occurs, which is inaccessible to plants [51]. The soil microbial community plays a crucial role in sulfur transformation. Microbes undertake processes such as mineralization, immobilization, oxidation and reduction of elemental sulfur and other reduced forms. The key step in this transformation is oxidation, conducted by microorganisms to convert sulfur into sulfate, which is accessible to plants. Chemolithotrophic bacteria, specifically those of the genus *Thiobacillus*, are particularly significant in facilitating this process [52,53,54].

In this study, we examined reactions to salinity and cadmium stress in *Tripolium pannonicum* after growth medium inoculation with sulfur-oxidizing bacteria belonging to the *Halothiobacillus* genus (previously belonging to the genus *Thiobacillus*). As an inoculant, we chose *H. halophilus*, which apart from its ability to oxidize sulfur, is characterized by salinity tolerance [54], which was crucial in our experiment. *T. pannonicum* was selected as the studied plant species due to its recognition as a model plant for investigating the tolerance to abiotic stress [42,44].

We evaluated plant responses by monitoring changes in growth and several biochemical parameters (content of elements, photosynthetic pigments, phenolic compounds, MDA, proline) reflecting plants’ physiological state and/or disorders caused by implemented stress. To summarize, our hypothesis, which required verification, was that soil inoculation with *Halothiobacillus halophilus* DSM 6132 (provided by ATCC) would increase the pool of sulfur available to plants and thus cause changes in the physiological and biochemical response of plants to applied stresses. We assumed that inoculating the substrate intended for growing plants with sulfur-oxidizing bacteria would increase the tolerance of *Tripolium pannonicum* to the salt and cadmium stress.

There are reports indicating that halotolerant plant-growth-promoting bacteria are emerging as an effective strategy for alleviating the adverse effects of high salinity. They contribute to enhancing the growth, development and yield of glycophytic plants and remediating degraded saline soils. These strategies involve various mechanisms, such as maintaining the ion balance, enhancing nutrient availability and promoting the biosynthesis of secondary metabolites, osmoprotectants, growth hormones and volatile organic compounds [39].

In our investigations, halotolerant sulfur-oxidizing bacteria promoted sea aster growth (stem length and GTI increment) under cadmium stress conditions applied to the substrate in the form of cadmium chloride (CdCl_2_). Surprisingly, we did not observe a clear positive effect of bacterial inoculation on control plants, which is in contradiction with documented observations by other authors. For example, the results obtained by Ali et al. [55] indicated that wild-type endophytes *Pseudomonas fluorescens YsS6* and *Pseudomonas migulae 8R6* accelerated tomato growth both under stressful conditions and in a non-stressed control. Eom et al. [56], using simple microbiological toxicity tests based on growth inhibition, respiration and bioluminescence measurements, investigated the effect of SOB in direct-contact tests to assess the toxicity of various metals. As the end point of toxicity assessment, the authors used the oxygen consumption by SOB, which acted as a universal indicator of SOB activity, regardless of soil properties. The results indicated that oxygen consumption by SOB in the tested soils spiked with arsenic, nickel, hexavalent chromium or zinc turned out to be smaller compared to soils spiked with mercury, copper, lead or cadmium. The authors suggested that soils slightly contaminated with Cd^2+^ ions are not very toxic to SOB. In our investigation, we similarly observed no evidence indicating that cadmium ions inhibit SOB activity. Furthermore, considering previous findings [8], it is possible to speculate that certain populations of *T. pannonicum* may exhibit enhanced growth under low concentrations of cadmium. This phenomenon can be explained by the hormensis effect, that is, so-called hormetic stimulation caused by low-dose stressor [57,58]. A possible explanation is also that *Halothiobacillus* produces certain substances, such as auxins, cytokinins or gibberellins, that directly or indirectly stimulate plant growth. It is likely, in this study, that the simultaneous application of salinity and cadmium stress was too strong for the bacteria to reduce plant growth inhibition.

The sulfur contents in roots of the tested plants increased after SOB application in all four variants of the reported experiment. This provides a full basis for the conclusion that the strain DSM 6132 of *H. halophilus* effectively carried out sulfur oxidation and thus increased the absorption of this element by plants. In a recently published work by Joshi et al. [53], the authors confirmed that SOB can be used to increase the soil fertility and sulfate production. Meanwhile, quite some time ago, Grayston and Germida [31] showed that fourteen isolates of sulfur-oxidizing bacteria, which they studied, oxidized sulfur to sulfate and thus increased the area of canola leaves, and seven of these isolates increased root and pod dry weights at maturity. The shoot material from canola inoculated with two of these isolates contained more iron, sulfur and magnesium than uninoculated canola. Other studies showed that the role of S (or its source) is also very important in the regulation of the Na^+^/K^+^ balance [59,60]. Further, the PCA results indicated that, in our study, the sulfur content was negatively correlated with changes in sodium content and chloride in plant tissues, but the addition of SOB did not change the level of Na in the leaves and reduced the Na content in the roots of plants treated with NaCl. When it came to chloride, we observed a lower accumulation of chlorine in the tissues of plants treated with CdCl_2_ after SOB inoculation, which was compatible with the sulfur level increase under the same conditions. Hidri et al. [35] described that inoculation with *Pseudomonas* sp. and *Glutamicibacter* sp. resulted in significantly higher shoot dry weight and less accumulation of Na^+^ and Cl^−^ in the upper parts of the plants of the halophyte *S. fruticosa* under salinity. Interestingly, an improvement in the growth parameters in our tested species was achieved after soil SOB inoculation only using the CdCl_2_ treatment, although the Cd content in the shoots did not decline in this variant of the experiment.

In our experiment, the contents of photosynthetic pigments in plants after soil bacterial inoculation were modified similarly to in the research of other authors; however, we did not find the same trend as others. We observed a decrease in chlorophylls and carotenoids in control plants and an increase in plants treated with cadmium. Photosynthetic pigments’ increment in CdCl_2_-stressed plants after SOB inoculation was related to the sulfur content increase in the leaves of stress-treated plants. This may have been related to the fact that sulfur is a component of chlorophylls’ biosynthetic and photosynthetic enzymes, and since the chlorophyll content of leaves is regulated by sulfur [50]. In Carreiras’s [34] research on *Halimione portulacoides*, both uninoculated and inoculated plants showed significant pigment changes under salt treatments. Szymańska et al. [61] found that halotolerant endophytes (*Pseudomonas stutzeri* ISE12 and *Kushneria marisflavi* CSE9) positively affected chlorophyll levels, leaf morphology, water retention, root growth and biomass accumulation in *Hordeum vulgare*, *Lactuca sativa* and *Helianthus annuus* under salt stress. They suggested that introducing halotolerant bacteria to crops could alleviate salt stress and enhance growth, emphasizing the importance of strain compatibility and validating universal plant stress indicators. In turn, another study showed that the *Zhihengliuella* halotolerans strain improved the content of total chlorophyll, carotenoids and total dry biomass in the halophyte *Haloxylon aphyllum* under salinity stress [62]. Regardless of SOB inoculation, the chlorophyll content was positively correlated with the carotenoid level and growth tolerance index of roots and leaves of *T. pannonicum*, but it had a negative interaction with dry weight, proline and phenolic compounds.

Our study indicates that cadmium tolerance in *T. pannonicum* can be associated with an increased level of phenolic compounds. Phenylpropanoids and flavonols increased pararelly with the sulfur content increase after SOB inoculation, both in root and above-ground tissue. This may have been due to the activation of certain synthesis pathways of phenolics or the production of some specific secondary metabolites by the bacterial strain itself, which then stimulated the synthesis of specific phenolic compounds in plants. There are no reports of the production of secondary metabolites by *Halothiobacillus* bacteria; however, there are known reports where halotolerant bacteria produce a secondary metabolite maintaining plant growth under saline environments, and these metabolites are only formed under abiotic stress [63,64,65]. These metabolites can aid plants through a diverse array of biochemical, physiological and molecular responses. They contribute to maintaining the ionic balance through Na^+^/K^+^ transporters, as well as enhancing water retention capacity, together with acting as osmoprotectants, antioxidants and compatible solutes [66,67]. Surprisingly, in our study, a similar mechanism was not observed in the case of salinity stress—SOB inoculation did not affect the content of phenolic compounds, but antiradical activity was enhanced. So, other molecules had to be responsible for this action, for example, proline, the content of which in leaves was strongly correlated with Na^+^ and Cl^−^ levels resulting in dry weight shifts. Regarding changes in the activity of the antioxidant system in plants after soil inoculation with bacteria, there was a report where halotolerant bacterial strains were able to reduce the contents of ascorbic acid, flavonoids, total phenols, proline, and malondialdehyde, along with catalase activity, and ultimately improve the antioxidant capacity of the halophyte *H. aphyllum* [62]. Hidri et al. [35] similarly reported that halophyte (*Suaeda fruticosa*) inoculation by *Glutamicibacter* sp. significantly reduced the MDA concentration under 600 mM NaCl. In our investigations, we also noted an MDA reduction after using the bacterial strain. Carreiras et al. [34] showed that plant-growth-promoting rhizobacteria (PGPR) improved the antioxidant response and promotion of osmotic balance traits in the halophyte *Halimione portulacoides*, which boosted the ability to cope with mild salt stress in this species. All these changes were in line with the differential elemental profiles (Na, K and Ca) observed in the different plant tissues.

The novel observation that various parameters reflecting tolerance mechanisms in the halophyte are influenced by soil inoculation with sulfur-oxidizing bacteria warrants further investigation.

## 4. Materials and Methods

### 4.1. Experimental Design

*Tripolium pannonicum* seed samples were obtained from the La Albufera Natural Park seed bank in Valencia, Spain. Seeds of equal size were surface sterilized with a mixture of 30% hydrogen peroxide and 70% ethanol (1:1; *v*/*v*) for 7 min, and then rinsed six times with sterile distilled water. Next, 10 seeds were sown into each individual 1 L pot (Ø = 11 cm) filled with a sterile mixture of sand and vermiculite 1:1 (*v*/*v*) (previously autoclaved in foil bags for 20 min at 121 °C under 0.1 MPa pressure). The pots were placed in a plastic tray, with a total of 48 pots prepared (6 pots per tray). The seeds were kept under controlled conditions (temperature: 24 ± 2 °C, 70% relative humidity and light/dark: 16/8 h) and were irrigated once per week with Hoagland medium [68]. After germinating the seeds, representative seedlings of the same size were selected and transferred into new pots with the same type of substrate as for the germination phase (1 seedling per pot). The seedlings grew in the same greenhouse conditions as when the seeds were germinated.

Six weeks after transferring the seedlings, the stress treatments were initiated. Control plants were watered once per week for 6 weeks by adding 1.5 L of Hoagland’s nutrient solution to each tray containing 6 pots. Salt and cadmium stress was applied by watering the plants in the same way but using 100 mM NaCl solutions, 1 mM CdCl_2_ and a mixture of 100 mM NaCl + 1 mM CdCl_2_, respectively.

Three weeks after the initiation of stress application, two variants were used for each treatment: (1) SOB inoculation (sulfur-oxidizing bacterial strain *Halothiobacillus halophilus* DSM 6132) and (2) a control treatment without the bacterial strain (non-SOB). Inoculation was repeated after 3 weeks. Nine weeks after the onset of stress, plants were harvested (separately above-ground parts and roots) for biochemical analyses.

#### Bacterial Strains

*Halothiobacillus halophilus* DSM 6132 was provided by ATCC (Manassas, VA, USA), and ATCC^®^ 4987 was used to inoculate the substrate. The bacteria were grown at 30 °C on a specific culture medium described as ATCC medium 1846: *Thiobacillus halophilus* medium (Supplement S1). After at least 72 h of culture incubation at 30 °C, the *H. halophilus* inoculum was prepared using cells suspended in 2% NaCl solution, which was then diluted to OD = 0.5 (OD—optical density, measured at 600 nm, equivalent to 1.5 108 cfu/mL). Next, 10 mL of bacterial inoculum (108 cfu/mL) was added to each pot twice, i.e., in the 3rd and 6th weeks of the experiment. The bacterial cell number was determined by using the plate count method.

To confirm the presence of bacteria in the substrate with cultivated plants, at the end of the experiment, bacteria were isolated from the soil according to the procedure described in Supplement S2.

### 4.2. Plant Growth Parameters

At the end of the experiment, the plant material was collected, and the following growth parameters were measured: stem length (SL), fresh weight (FW) (Supplement S3) and dry weight (DW) (Supplement S3) of above-ground parts and roots separately. To obtain the dry weight (DW), a fraction of the fresh material was weight and dried at 105 °C until a constant weight was achieved. DW% was calculated for the shoots and roots of each plant as follows:

$$\mathrm{DW}\%=(\frac{DW}{FW})\cdot 100$$

The stress tolerance index (GTI) was calculated for the shoot dry biomass using the following formula:

GTI = (mean DW of treated shoots/mean DW of non-treated shoots) × 100

### 4.3. Concentrations of Sulfur, Sodium, Chloride and Cadmium in Plants

For determination of the total metal content, the dried plant tissue samples were digested with a 9 mL mixture (1:3 *v*/*v*) of concentrated acids (HCl and HNO_3_) using the wet method in a closed system in a microwave oven (Multiwave 3000, Anton Paar, Austria). The digestion was carried out in accordance with the program of the following parameters: power 1400 u/min, temperature 240 °C, time to reach the maximum power 5 min, time of maximum power 15 min, ventilation time 5 min, cooling time 40 min. Concentrations of elements were determined using a Perkin-Elmer model Optima 7300 DV inductively coupled plasma atomic emission spectrophotometer (ICP-OES). Each sample of the plant material was analyzed in two replicates. If the analysis results of those replications differed from one another by more than ±5%, another two analyses of that sample were conducted. The quality of the determinations was verified based on the results from heavy metal determinations obtained for the internal standard and on the certified reference material CRM023-050—Trace Metals—Sandy Loam 7 (Supelco, Bellefonte, PA, USA).

### 4.4. Plant Biochemical Analyses

For biochemical analyses, plant material was preserved by grinding in liquid nitrogen and storing at −80 °C. All measurements were taken in three replicates (unless stated otherwise).

#### 4.4.1. Photosynthetic Pigments

Chlorophyll and carotenoid concentrations were determined spectrophotometrically in 80% acetone extracts. The absorbance was measured at 470, 646 and 663 nm and concentrations of the respective pigments were calculated according to Wellburn [69].

#### 4.4.2. Proline

Proline (Pro) content was assayed according to Bates et al. [70] with minor modifications. Tissue samples were homogenized in 3% aqueous sulfosalicylic acid and centrifuged (4 °C; 15 min; 3000× *g*). The extracts supplemented with acid-ninhydrin and glacial acetic acid (1:1:1) were incubated in a boiling water bath for 1 h. The reaction was terminated in an ice bath. The reaction mixture was extracted with toluene by vigorous mixing and absorbance of the toluene phase was read at 520 nm (Genesys 10 spectrophotometer, ThermoFisher Scientific, Waltham, MA, USA). Calculation of Pro content was performed on the basis of the standard curve.

#### 4.4.3. Phenolic Compounds

Total phenolics were determined with the Folin–Ciocalteu (F–C) assay [71]. First, the plant material was homogenized in ice-cold 80% methanol and centrifuged (3000× *g* for 15 min at room temperature). Incubation of the methanolic extract with 10% F–C reagent (Sigma-Aldrich, Poznań, PL, USA) and 700 mM Na_2_CO_3_ lasted for 2 h. Absorbance was measured at 765 nm immediately after the incubation. Total phenolic content was expressed as chlorogenic acid equivalents.

#### 4.4.4. Phenolic Profile

Phenolic compounds (total phenolic content—TPC, phenylpropanoids, flavonols and anthocyanins) were determined using UV/VIS spectrophotometry [72]. Chlorogenic acid (CGA), caffeic acid (CA), quercetin (QC) and cyanidin (CY) (Sigma-Aldrich) were used as standards for TPC, phenylpropanoids, flavonols and anthocyanin content, respectively. Samples were ground with 1 mL of 80% methanol and centrifuged for 15 min at 3000× *g* and room temperature. The supernatant (0.25 mL) was mixed with 0.25 mL 0.1% HCl (in 96% ethanol) and 4.50 mL 2% HCl (in water) and, after 30 min incubation, the absorbances at 280, 320, 360 and 520 nm were read (U-2900 spectrophotometer, Hitachi High-Tech, Tokyo, Japan). The content of phenolic compounds was expressed in mg of respective standard equivalents per 100 g of FW.

#### 4.4.5. Radical Scavenging Activity with DPPH Radical

Stable free radical 2,2-diphenyl-1-picrylhydrazyl (DPPH, Sigma-Aldrich) was used to test radical scavenging activity (RSA) in shoot samples [73]. The changes in absorbance (recorded with Hitachi U-2900 spectrophotometer, Japan) of 0.1 mM DPPH˙ solution (2.95 mL), as a result of DPPH˙ neutralization, were measured at 517 nm after 30 min incubation with 50 µL of plants extracts (80% methanolic supernatants obtained as described for phenolic determination). The antioxidant activity of the extracts was expressed as the percentage of DPPH radical neutralized by the plant extract.

#### 4.4.6. Lipid Peroxidation

MDA (malondialdehyde), a final product of membrane lipid peroxidation and a reliable marker of oxidative stress [74], was determined as described by Hodges et al. [75]. Briefly, 80% methanol extracts were mixed with 0.5% thiobarbituric acid (TBA) in 20% TCA, and with 20% TCA without TBA for the controls, and then incubated at 95 °C for 20 min. After stopping the reaction on ice, the supernatant’s absorbance was measured at 532 nm. The non-specific absorbance at 600 and 440 nm was subtracted, and the MDA concentration was calculated with the equations described in Hodges et al. [75].

### 4.5. Statistics

Data were statistically analyzed using Statistica 13.0 software (Tibco, Palo Alto, CA, USA). Two-way ANOVA and post hoc Tukey’s testing were used to assess differences between *T. pannonicum* responses to stress and bacteria treatments. Principal component analysis (PCA) was performed to visualize relationships between biochemical parameters and plant treatments.

## 5. Conclusions

Our study has revealed that sulfur-oxidizing bacteria of the *Halothiobacillus halophilus* species induce a distinct physiological reaction in *Tripolium pannonicum* plants exposed to salinity and heavy metal stresses in comparison with non-inoculated plants. Although the physiological responses of examined plants were miscellaneous, we have identified changes in growth and biochemical parameters in the plants after the application of sulfur bacteria in conjunction with salt and cadmium stress. SOB inoculation has prevented hampering the growth, and GTI as well as photosynthetic pigments decrease in plants exposed to cadmium. We have found that the sulfur content interacts with the levels of phenylpropanoids and flavonols (high increase) and that these parameters may be related to cadmium tolerance in *T. pannonicum*. The results indicated that lipid peroxidation occurring after separate applications of salt stress and cadmium stress was alleviated when a bacterial inoculum was used. When comparing non-inoculated and inoculated plants, a notable enhancement in cadmium stress tolerance was observed as opposed to salt stress; thus, we verified our hypothesis that substrate inoculation with sulfur-oxidizing bacteria causes a change in the response of plants to salt and cadmium stress. Our study sheds light on the beneficial role of sulfur-oxidizing microorganisms in changing the response of halophytes to cadmium. In the future, it is necessary to extend research with sulfur-oxidizing bacteria, to determine their specific function in modulating plant responses to abiotic stress.

## Figures and Tables

**Table 1:** Growth parameters in *T. pannonicum* subjected to sodium chloride (100 mM NaCl), cadmium chloride (1 mM CdCl_2_), a mixture of sodium chloride and cadmium chloride (100 mM NaCl + 1 mM CdCl_2_) and sulfur-oxidizing bacteria. DW%—dry weight content (%), GTI%—growth tolerance index, SOB—*Halothiobacillus halophilus*-inoculated substrate, non-SOB—non-inoculated substrate.

		DW%		GTI%		Stem Length [cm]	
		Non-SOB	SOB	Non-SOB	SOB	Non-SOB	SOB
leaves	control	9 a	10 B	100 c	100 B	60 c	49 D
	NaCl	27 b	19 D *	122 c	121 B	11 a	14 B
	CdCl_2_	8 a	7 A	32 a	90 AB *	22 b	41 C *
	NaCl + CdCl_2_	48 c	14 C *	54 b	78 A	25 b	8 A
roots	control	12 c	7 B *	100 d	100 C		
	NaCl	6 b	5 AB	49 c	58 B *		
	CdCl_2_	9 b	20 C *	23 b	75 B *		
	NaCl + CdCl_2_	3 a	4 A	10 a	13 A		

Different lowercase letters indicate significant differences between plants cultivated on substrate non-inoculated by SOB within different stress treatments. Different capital letters indicate statistically significant differences between plants cultivated on substrate inoculated by SOB within different stress treatments. * indicates statistically significant differences between non-inoculated and inoculated plants within the same stress treatment, according to Tukey’s test (α = 0.05), *n* = 5.

**Table 2:** Total contents of phenols, phenylpropanoids, flavonols and anthocyanins (mg·g^−1^ f.w.) in *T. pannonicum* subjected to sodium chloride, cadmium chloride and sulfur-oxidizing bacteria. SOB—substrate inoculated with *Halothiobacillus halophilus*, non-SOB—substrate not inoculated with *H. halophilus*.

	Total Phenolics		Phenylpropanoids		Flavonols		Anthocyanins	
	Non-SOB	SOB	Non-SOB	SOB	Non-SOB	SOB	Non-SOB	SOB
control	18.5 a	55.5 B *	15.9 a	19.8 B	5.3 a	9.7 B *	0.7 a	0.7 A
NaCl	54.2 b	50.5 B	19.6 b	23.3 B	10.7 b	11.8 C	0.9 a	1.4 B *
CdCl_2_	8.0 a	23.9 A *	4.5 a	10.7 A *	3.5 a	6.6 A *	0.7 a	0.7 A
NaCl + CdCl_2_	56.7 b	50.8 B	22.0 c	28.8 C *	30.6 c	20.3 D *	2.5 b	1.3 B *

Different lowercase letters indicate significant differences between plants cultivated on substrate non-inoculated by SOB within different stress treatments. Different capital letters indicate statistically significant differences between plants cultivated on substrate inoculated by SOB within different stress treatments. * indicates statistically significant differences between inoculated and non-inoculated plants within the same stress treatment, according to Tukey’s test (α = 0.05), ±SE, *n* = 5.

## Data Availability

Data are contained within the article and Supplementary Materials.

## Supplementary Materials

The following supporting information can be downloaded at https://www.mdpi.com/article/10.3390/ijms25052455/s1.
