# Supplementary material for: Sulfur-Oxidizing Bacteria Alleviate Salt and Cadmium Stress in Halophyte Tripolium pannonicum (Jacq.) Dobrocz
Source: Int J Mol Sci. 2024 Feb 20;25(5):2455. doi: 10.3390/ijms25052455 (PMC10931265; doi:10.3390/ijms25052455)
Supplement: Supplementary file 1 [file ijms-25-02455-s001.zip › Supplement 3.docx]

|  |  | **FW [g]** | | **DW [g]** | |
| --- | --- | --- | --- | --- | --- |
|  |  | **Non-SOB** | **SOB** | **Non-SOB** | **SOB** |
| leaves | control | 1,177 | 0,937 | 0,103 | 0,098 |
|  | NaCl | 0,460 | 0,643 | 0,126 | 0,119 |
|  | CdCl_2_ | 0,397 | 1,213 | 0,033 | 0,089 |
|  | NaCl + CdCl_2_ | 0,116 | 0,526 | 0,056 | 0,076 |
| roots | control | 0,993 | 0,963 | 0,116 | 0,065 |
|  | NaCl | 0,863 | 0,840 | 0,057 | 0,038 |
|  | CdCl_2_ | 0,313 | 0,223 | 0,027 | 0,049 |
|  | NaCl + CdCl_2_ | 0,387 | 0,253 | 0,012 | 0,074 |

**Supplement 3.** Fresh weight (FW) and dry weight (DW) in *T. pannonicum* subjected to sodium chloride (100 mM NaCl), cadmium chloride (1 mM CdCl_2_), a mixture of sodium chloride and cadmium chloride (100 mM NaCl + 1 mM CdCL_2_) and sulfur-oxidizing bacteria (SOB).
