# Supplementary material for: Sulfur-Oxidizing Bacteria Alleviate Salt and Cadmium Stress in Halophyte Tripolium pannonicum (Jacq.) Dobrocz
Source: Int J Mol Sci. 2024 Feb 20;25(5):2455. doi: 10.3390/ijms25052455 (PMC10931265; doi:10.3390/ijms25052455)
Supplement: Supplementary file 1 [file ijms-25-02455-s001.zip › Supplement 1 .docx]

**ATCC medium: 1846 *Thiobacillus halophilus* medium**

***Solution A:***

Na2HPO4 ........................................6.3 g

KH2PO4 .........................................1.5 g

Distilled water..............................100.0 ml

Autoclave at 121C for 15 minutes.

***Solution B:***

NaCl..........................................50.0 g

Na2S2O3 . 5H2O…...............................5.0 g

NH4Cl..........................................0.4 g

Modified Hutner's Basal Salts (see below).....20.0 ml

Phenol red....................................10.0 mg

Agar, Noble (BD 214230).......................15.0 g

Distilled water..............................900.0 ml

Adjust medium, if necessary, to final pH 7.3 with NaOH. Autoclave at

121C for 15 minutes.

***Modified Hutner's Basal Salts:***

Nitrilotriacetic acid.......10.0 g

MgSO4 . 7H2O ................29.7 g

CaCl2 . 2H2O .................3.34 g

Ammonium molybdate...........9.25 mg

FeSO4 . 7H2O ................99.0 mg

Metals "44" (see below).....50.0 ml

Distilled water to...........1.0 L

Dissolve and neutralize the nitrilotriacetic acid with KOH (7.3 g); add the other ingredients and adjust the pH to 6.6 - 6.8 before bringing the volume to 1.0 L with distilled water.

***Metals "44":***

EDTA.........................0.25 g

ZnSO4 . 7H2O .................1.1 g

FeSO4 . 7H2O .................0.5 g

MnSO4 . 7H2O .................0.154 g

CuSO4 . 5H2O .................0.04 g

Co(NO3)2 . 6H2O...............0.025 g

Na2B4O7 . 10H2O...............0.018 g

Distilled water............100.0 ml

Initially add a few drops of H2SO4 to the distilled water to retard precipitation.
