# Supplementary material for: Sulfur-Oxidizing Bacteria Alleviate Salt and Cadmium Stress in Halophyte Tripolium pannonicum (Jacq.) Dobrocz
Source: Int J Mol Sci. 2024 Feb 20;25(5):2455. doi: 10.3390/ijms25052455 (PMC10931265; doi:10.3390/ijms25052455)
Supplement: Supplementary file 1 [file ijms-25-02455-s001.zip › Supplement 2 .docx]

**Bacteria isolation from the soil**

1. Measure 100 ml of distilled water in a graduated cylinder and add it to a sterile bottle.

2. Weigh out 1 g of the soil sample and add it to the bottle of distilled water.

3. Tightly cap the bottle and shake it to thoroughly mix the solution.

4. Label four sterile test tubes “10^-3,” “10^-4,” “10^-5,” and “10^-6.”

5. Add 9 ml of distilled water to each of the tubes, using one of the pipettes.

6. Transfer 1 ml of the solution in the bottle to the tube labeled “10^-3,” using a new pipette.

7. Cap the tube and swirl it gently until the solution is well mixed.

8. Transfer 1 ml of the solution in the “10^-3” test tube to the “10^-4” tube with a new pipette.

9. Cap the “10^-4” tube and swirl to mix.

10. Repeat this method to transfer solution from the “10^-4” tube to the “10^-5” tube and then from the “10^-5” tube to the “10^-6” tube.

11. Plate three samples each from the “10^-4,” “10^-5” and “10^-6” tubes. Use a new pipette to transfer 1 ml of solution from the tube into a Petri plate.

12. Add about 15 ml of nutrient agar to the plate; then put the lid on the plate and swirl gently so that the agar covers the bottom of the plate.

13. Make a control plate by putting 1 ml of distilled water into a Petri plate, using a new pipette. Add agar; put the lid on and swirl the plate.

14. Leave the Petri plates upright until the agar has set. Then invert the plates and incubate them—either in an incubator or at room temperature—for as little as 24 hours and up to five days.

15. Remove the plates from the incubator after the desired amount of incubation time.

16. Count the bacterial colonies on plates containing about 30 to 300 colonies. Use a permanent marker to mark the colonies you have already counted in order to avoid counting the same colonies twice.

17. Divide the number of colonies counted by the dilution - “10^-4,” “10^-5” or “10^-6”—of the soil solution for each plate.

18. Find the number of cultivatable bacteria in the original gram of soil by averaging the results from each countable plate.
